# Comparison of the bioavailability of benzo[a]pyrene (B[a]P) in a B[a]P-contaminated soil using the different addition approaches

**DOI:** 10.1038/s41598-019-40813-1

**Published:** 2019-03-07

**Authors:** Xinxin Ye, Jingjing Ma, Junling Wei, Kai Sun, Qizhong Xiong

**Affiliations:** Anhui Province Key Laboratory of Farmland Ecological Conservation and Pollution Prevention, School of Resources and Environment, Anhui Agricultural University, Hefei, 230036 China

## Abstract

Determination of the bioavailability of the hydrophobic organic contaminant benzo[a]pyrene (B[a]P) is extremely important for assessing its environmental risk. The effect of addition manner of B[a]P on the bioavailability and toxicity of B[a]P in soil remains unclear. In this study, soil samples, spiked with B[a]P by one-time or multiple-time additions, were tested to investigate the available fraction of B[a]P in soils, the uptake of B[a]P by red wiggler worms (*Eisenia fetida*), as well as superoxide dismutase (SOD) and peroxidase (POD) activities in earthworm coelomocytes at different periods. Results showed that the available fraction of B[a]P in soils and the amount of B[a]P assimilated by earthworms declined sharply from 1 d to 28 d during the incubation period and then decreased slowly from 28 to 56 d in both the one-time and the multiple-time addition tests. The available fraction of B[a]P in soils and its uptake by earthworms were significantly lower in multiple-time addition samples than those in one-time addition samples, a finding which was consistent with the SOD and POD activities in earthworms during the whole 56-d incubation period. These variations in the characteristics of the two addition treatments may be due to the differences in the way the B[a]P aged in the soil. These results indicated that the addition method was an important factor influencing the bioavailability of organic contaminants in soils.

## Introduction

Polycyclic aromatic hydrocarbons (PAHs) are degradation-resistant and hydrophobic organic pollutants, posing a serious threat to human health due to their carcinogenic, mutagenic, and teratogenic natures^1,2^. The contamination of PAHs in soil has aroused increasing concerns in many countries, because the soil is the primary storage pool of PAHs^3,4^. The most extensively studied compound among PAHs is benzo[a]pyrene (B[a]P), because B[a]P is considered to be one of the most carcinogenic pollutants among the PAHs^5^. Moreover, B[a]P has a structure of five rings with a high octanol-water partition coefficient (*K*_ow_ ≈ 1 × 10^6^), which can be easily adsorbed onto soil particles and assimilated by soil organisms^6^. Therefore, it is extremely important to accurately evaluate the toxicity of B[a]P in soils.

At present, most studies on the eco-toxicological effects of B[a]P are conducted under simulated conditions in the laboratory, in which the B[a]P is introduced into the soil at one time^7,8^. However, in practical soil-contamination conditions, the concentrations of B[a]P in soils reach a certain level through the pattern of multiple-time accumulation^9^, in which the available fraction of B[a]P in the soil may be different from those used in the conventional tests, with one-time addition. Therefore, the addition method by which the pollutant was added to the soil needs to be considered in the risk assessment of B[a]P-contaminated soils^10,11^.

The responses of earthworms to sub-chronic exposure to pollutants, often referred to as early-warning signals, could, in fact, provide information important for assessing the ecotoxicological risk of pollutants^12–14^. Many reports have shown that PAHs can induce an increase in reactive oxygen species (ROS) in earthworms^15,16^. The defense against ROS in earthworms consists of ROS-scavenging enzymes, such as superoxide dismutase (SOD) and peroxidase (POD). Superoxide dismutase is responsible for the transformation of O_2_^−^ into H_2_O_2_, while POD can degrade peroxides such as H_2_O_2_^17^. Therefore, alterations in the biomarkers of toxicology in earthworms reflect oxidative damage to the whole organism, caused by the pollutants and their metabolites. However, little information is known about the effect of B[a]P spiked in different methods on its bioavailability in soils and on antioxidants in earthworms.

The main aims of this study were: (1) to investigate the variations in the available fraction of B[a]P in soil, and the quantities of B[a]P accumulated in earthworms (*Eisenia fetida*) in one-time and multiple-time addition treatments; (2) to compare the effects of B[a]P on SOD and POD activities in earthworm coelomocytes in the two addition methods; and (3) to examine the correlations between either SOD or POD activities and the available fraction of B[a]P in soil as well as the uptake of B[a]P in earthworms. The purpose was to obtain a more comprehensive understanding of the effects of B[a]P on earthworms when spiked using different addition methods, which might help to more accurately assess the risk of B[a]P in the soil environment.

## Results

### The available fraction of B[a]P in soil

**Figure 1:**
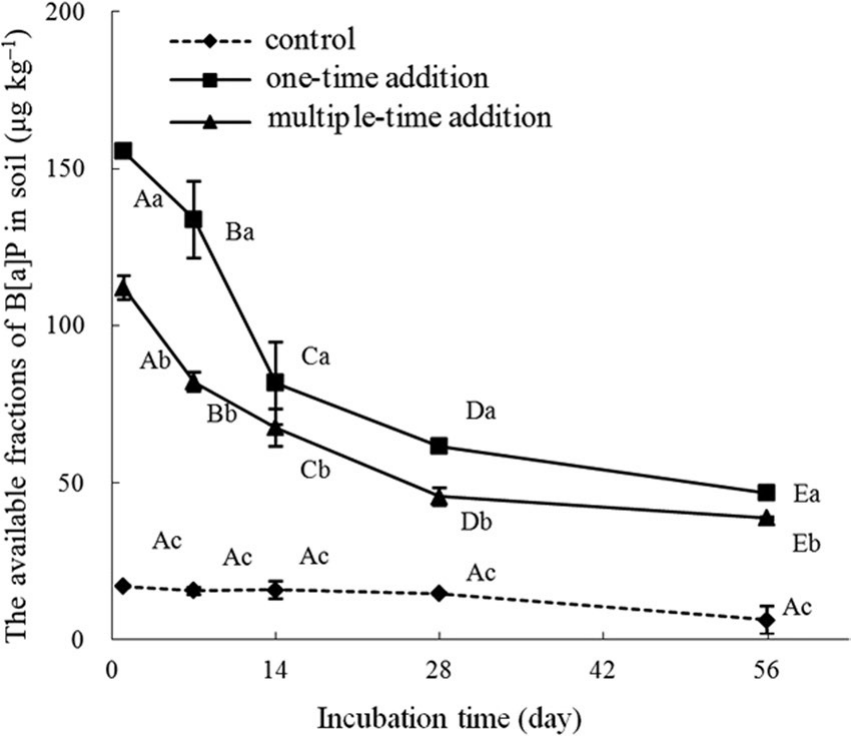
Changes in the available fraction of B[a]P in sterilized soil over time. Control represents treatments without the addition of B[a]P, one-time addition are treatments with the addition of B[a]P at one time, multiple-time addition are treatments with the addition of B[a]P by twelve times. Error bars represent standard deviation, and samples with a common upper-case letter indicate no significant difference among different incubation times under the same treatment (P > 0.05, n = 3), and those with a common lower-case letter indicate no significant difference among different treatments at the same incubation time (P > 0.05, n = 3).

As shown in Fig. 1, the available fraction of B[a]P in the control samples during the 56-d incubation period were statistically unchanged. As incubation time progressed, the amount of available B[a]P in soil sharply decreased between 1 d and 28 d, and then slowly declined between 28 d and 56 d under both one-time and multiple-time addition treatments (Fig. 1). In the one-time addition treatment, the available fraction declined by 14% on day 7, and declined by 64% on day 56, compared to day 1. The corresponding figures were 29% on day 7 and 62% on day 56 in the multiple-time addition treatment.

The available B[a]P content in soil in the multiple-time treatment was significantly lower (P < 0.05) than that in the one-time addition treatment at each incubation date (Fig. 1). The available B[a]P content in the multiple-time treatment was 72% and 76% of that in the one-time addition treatment on day 1 and day 56, respectively. These results showed that the one-time addition treatment resulted in B[a]P being easier to be desorbed from soil than in the multiple-time addition treatment.

### B[a]P assimilation in earthworms

**Figure 2:**
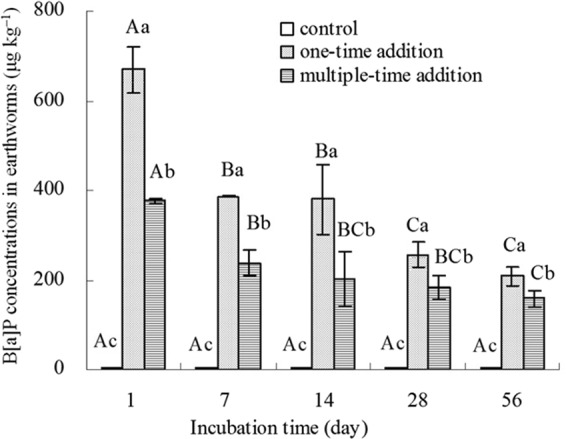
B[a]P assimilation in earthworms. Control represents treatments without the addition of B[a]P, one-time addition are treatments with the addition of B[a]P at one time, multiple-time addition are treatments with the addition of B[a]P by twelve times. Error bars represent standard deviations. Samples with a common upper-case letter indicate no significant difference among different incubation times under the same treatment (P > 0.05, n = 3), and those with a common lower-case letter indicate no significant difference among different treatments at the same incubation time (P > 0.05, n = 3).

There were no significant variations in the uptake of B[a]P by earthworms in the control samples as time progressed (Fig. 2). In the one-time addition treatment, the concentration of B[a]P in earthworms was highest on day 1, and was significantly higher (P < 0.05) than that at all later times, and there were also significant differences in the B[a]P concentration in earthworms between 14 d and 28 d (Fig. 2). The uptake of B[a]P by earthworms also showed significant declines as incubation period increased in the multiple-time addition treatment, being highest on day 1 and lowest on day 56 (Fig. 2). Compared to day 1, the earthworm uptake of B[a]P decreased by 32% on 56 d in the one-time addition treatment, while the corresponding value was 45% in the multiple-time addition treatment.

**Figure 3:**
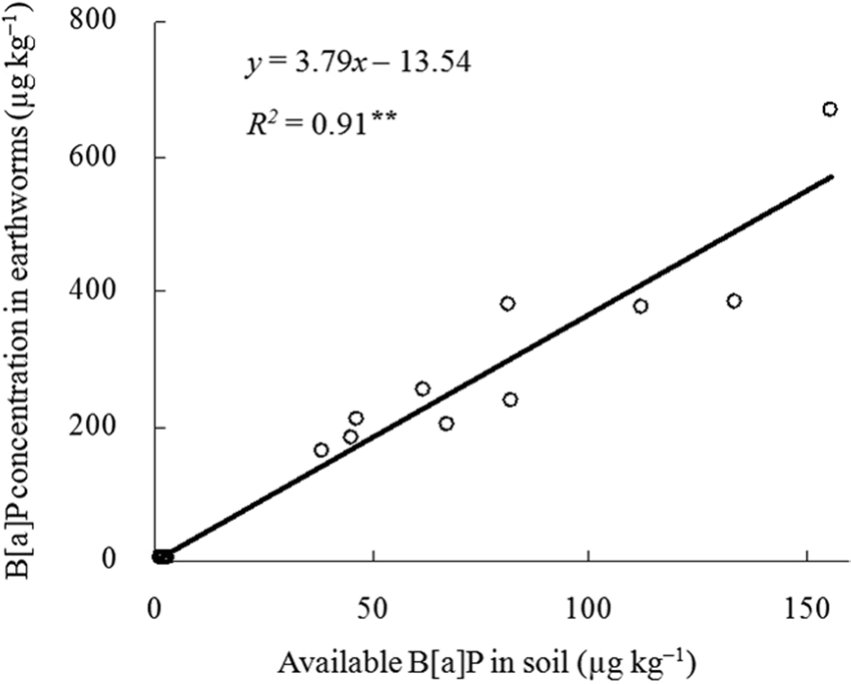
Correlation between the available B[a]P concentration in soil and the amounts of B[a]P assimilated by earthworms, ^**^represent significant relationship (P < 0.01, n = 15).

The earthworm uptake of B[a]P from soil contaminated by the one-time addition treatment was significantly higher (P < 0.05) at each stage than that from soil in the multiple-time addition treatment (Fig. 2). The concentrations of B[a]P in earthworms exposed to soil from the multiple-time treatment were 57% and 79% of those from the one-time addition treatment on days 1 and 56, respectively (Fig. 2). A significant positive correlation (P < 0.01) was detected between the concentration of B[a]P in earthworms and the available B[a]P concentration in soil (Fig. 3), suggesting that the uptake of B[a]P in earthworms reflected the availability of B[a]P in soil at different incubation periods. These results indicated that a relatively small decrease in desorption capacity of B[a]P in the multiple-time addition treatment leads to the low absorption of B[a]P by earthworms.

### SOD and POD activities in earthworm coelomocytes

**Figure 4:**
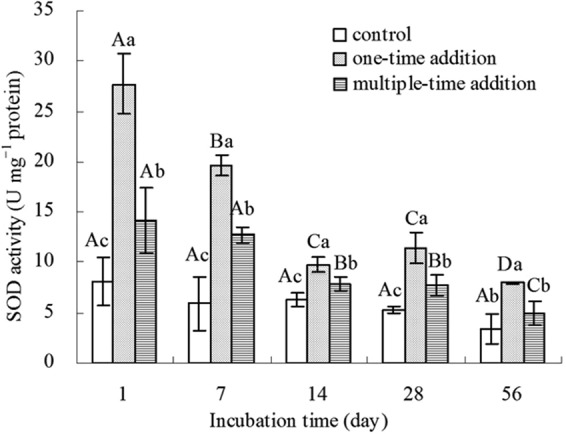
SOD specific activities in earthworms. Control represents treatments without the addition of B[a]P, one-time addition are treatments with the addition of B[a]P at one time, multiple-time addition are treatments with the addition of B[a]P by twelve times. Error bars represent standard deviations. Samples with a common upper-case letter indicate no significant difference among different incubation times under the same treatment (P > 0.05, n = 3), and those with a common lower-case letter indicate no significant difference among different treatments at the same incubation time (P > 0.05, n = 3).

The effect of B[a]P on the SOD activity of earthworms as a result of the two addition treatments is displayed in Fig. 4. The SOD activity in earthworms in the one-time addition treatment was significantly (P < 0.05) higher than that in the control at each stage. The same trend was observed in the multiple-time addition treatment except for the value after 56 d incubation, where the earthworm SOD activity in the multiple-time addition treatment was not significantly different from the control value (Fig. 4). SOD activity decreased in both B[a]P addition treatments as the duration of incubation increased. The SOD activity was significantly higher in the one-time addition treatment than that in the multiple-time addition treatment at all stages. The SOD activities in earthworm coelomocytes in the multiple-time addition treatment were 49% and 63% of those in the one-time addition treatment on day 1 and day 56, respectively.

**Figure 5:**
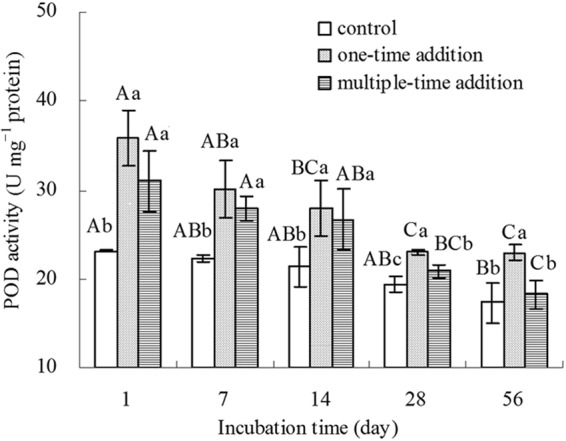
POD specific activities in earthworms. Control represents treatments without the addition of B[a]P, one-time addition are treatments with the addition of B[a]P at one time, multiple-time addition are treatments with the addition of B[a]P by twelve times. Error bars represent standard deviations. Samples with a common upper-case letter indicate no significant difference among different incubation times under the same treatment (P > 0.05, n = 3), and those with a common lower-case letter indicate no significant difference among different treatments at the same incubation time (P > 0.05, n = 3).

As shown in Fig. 5, the POD activity was significantly higher (P < 0.05) in earthworms in the one-time addition treatment at all stages than in the control group. In the multiple-time addition treatment, the POD activity was significantly (P < 0.05) higher than the control group from day 1 to day 28 d of the incubation period, but was not significantly higher than that of the control on day 56. There was no significant variation in the POD activity between the two addition-time treatments from day 1 to day 14 of incubation, whereas POD activity in the multiple-time addition treatment was lower than that in the one-time addition treatment after 14 days of exposure. The POD activity in the multiple-time treatment was 88% and 71% of those in the one-time addition treatment on days 28 and 56, respectively. To summarise, the manner in which the B[a]P was added to the soil had a significant effect on the SOD and POD activities in the earthworms.

**Figure 6:**
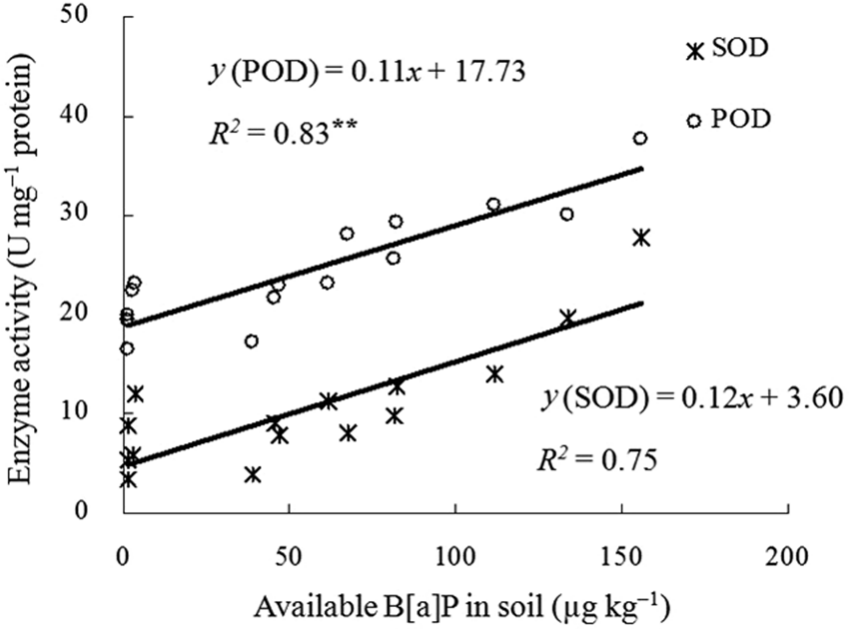
Correlations between SOD or POD activities and the available B[a]P concentration in soil, ^**^represent significant relationship (P < 0.01, n = 15).

**Figure 7:**
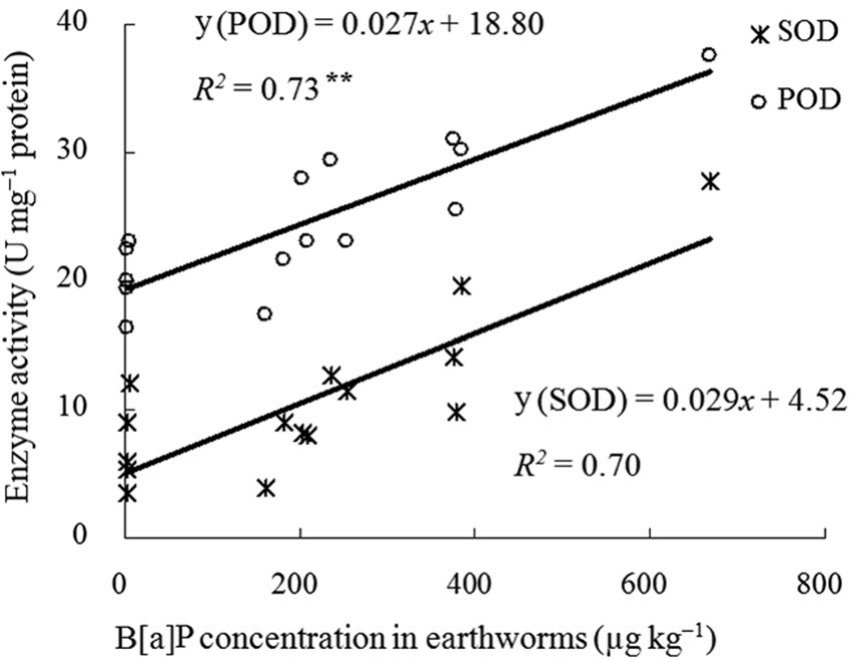
Correlations between SOD or POD specific activities and the B[a]P assimilated in earthworms, ^**^represent significant relationship (P < 0.01, n = 15).

SOD and POD activities in earthworm coelomocytes were significantly positively correlated with the concentration of available B[a]P in soil, with the correlation coefficients (*r*) being 0.87 and 0.91 (P < 0.01), respectively (Fig. 6). The SOD and POD activities in earthworm coelomocytes were significantly positively correlated (P < 0.01) with the amount of B[a]P absorbed by earthworms, and the correlation coefficients (r) were 0.84 and 0.85, respectively (Fig. 7). These correlation analyses revealed that the SOD and POD activities in earthworm coelomocytes were good biomarkers for the eco-toxicological effects of B[a]P, which were dependent on the manner in which the B[a]P was added to the soil.

## Discussion

B[a]P was more readily available at the beginning of the incubation in soil, but its availability decreased rapidly with prolonged contact time between soil and B[a]P, which was in agreement with previous studies on the bioavailability of pesticides and PAHs^10,18^. The phenomenon may be related to the aging process of B[a]P in soil. B[a]P is rapidly adsorbed onto soil solids such as soil particles and organic matter, and then slowly diffuses into soil aggregates and moves through micro-pores in the soil^19,20^.

In this present study, the concentration of available B[a]P in the soil and its concentration in earthworms in the multiple-time addition treatment were lower than the corresponding values in the one-time addition treatments at each stage of the incubation period. Because the microcosms were sterilized during the incubation course, the microbial degradation of B[a]P could be ignored^21^. The aging processes may cause the differences in B[a]P bioavailability in soils contaminated by the two different manners of addition of B[a]P. The proportion of the B[a]P distributed on organic matter and the diffusion of B[a]P into the micro-pores can lead to differences in release from the adsorbed sites and in assimilation by soil organisms^22^. The photodegradation and volatilization can not affect the B[a]P bioavailability in soil, because the experiment were carried out in a closed environment and the test time is short.

A role for SOM in reducing the bioavailability of aged B[a]P in soil has been proposed^10^. Aging of compounds in soil occurs due to adsorption on ‘rubbery’ or ‘glass-like’ regions of SOM via isocratic diffusion or partitioning coupled with hole-filling mechanisms^23^. The decreased level of bioavailability of B[a]P after aging is closely related to the amount of SOM. The concentrations of available PAHs (e.g. phenanthrene, pyrene) decreased significantly with increasing SOM^11^. Since there were more adsorption sites for B[a]P on SOM in the multiple-time addition treatment than in the one-time addition treatment, the transformation of more of the available fraction into organic matter-bound fraction results in less extractability and bioavailability of B[a]P in the multiple-time addition treatment.

The pathways by which B[a]P enters the soil affects the extraction rates^24^. Minute pores or voids are abundant in soils. Pores with diameters < 100 nm were present in all soils examined^25^, and pores or voids with diameters of 0.3–1.0 nm are also within the size ranges of organic molecules of toxicological significance^26^. We deduced that The amount of B[a]P added to the soil was relatively enormous, and readily saturated the pores in the one-time addition treatment, leading to a large proportion of B[a]P being unsteadily adsorbed onto soil particles. On the other hand, the available B[a]P fraction in the multiple-time addition treatment were partially converted into the bound fraction, locked in the micro-pores of the soil matrix or embedded into the reticular formation of humus, making it difficult to desorb the B[a]P. Therefore, in the multiple-time addition treatment, B[a]P entered into the deeper adsorption sites and was barely desorbed, leading to its decreased availability to soil organisms.

Finally, B[a]P can be easily entrapped within the solid phase of organic matter and also in the micro-pores or voids at specific sites in the soil matrix in the multiple-time addition treatment. The available fraction of B[a]P in soil and its absorption by earthworms in multiple-time addition was lower than that in the one-time addition treatment.

The present study showed that the concentration of the Tenax-TA adsorbed fraction of B[a]P in soil was positively (P < 0.01) correlated with the amount of B[a]P assimilated in earthworms, indicating that the Tenax-TA extractable fraction of B[a]P in soil was a good indicator of the bioavailability of B[a]P in the two addition treatments. Some researchers have also reported that the available fraction of PAHs, which could be extracted by Tenax-TA from soil, was positively related to the accumulated amounts of PAHs in organisms^18,27^. The distribution of organic pollutants between soil matrix and pore water, and the partitioning between pore water and organisms influences the extractability and bioavailability of contaminants in soil^28,29^. Extraction methods of B[a]P with Tenax-TA proved to be satisfactory, with direct correlation to the bioavailable fraction for both the one-time and the multiple-time addition methods.

Biochemical responses of organisms to environmental stress are regarded as early-warning indices of pollution in the environment^30,31^. Several indicators are routinely used to evaluate the toxic effects of B[a]P on earthworms, and antioxidant enzymes play important roles in the adaption of organisms to stressful conditions through the elimination of ROS^32^. In the present research, the activities of SOD and POD in earthworm coelomocytes were significantly (P < 0.05) lower in the multiple-time addition treatment than those in the one-time addition treatment. The reason for this might be that the available fraction of B[a]P in the multiple-time addition treatment was significantly lower than that in the one-time addition treatment. It has been accepted that the bioavailability of organic pollutants is one of the main factors influencing the extent of their toxicity towards soil biota^33,34^, while the decreased bioavailability of B[a]P in soil reduced the toxic effects of B[a]P to earthworms. These enzyme activities are significantly correlated to the bioavailability of B[a]P (the available fraction in the soil and the fraction taken up by earthworms) in the two addition manners. Thus, an alteration in earthworm SOD or POD activities can be regarded as an early index of environmental pollution.

## Conclusions

The available fraction of soil B[a]P in the multiple-time addition treatment was significantly lower (P < 0.05) than that in the one-time addition treatment, which was in agreement with the toxic effects of B[a]P to earthworms. The most likely explanations are the higher sorption of the B[a]P to the soil organic fractions, and the greater diffusion of the B[a]P into the micro-pores within the solid phases of the soil after multiple-time addition than after one-time addition. Physical and chemical processes reduced the availability of the B[a]P in soil and its transfer to earthworms in the multiple-time addition treatment. The results presented in this study suggest that the manner in which the PAH was added to the soil affected the bioavailability of B[a]P in soil, and needs to be considered in order to accurately evaluate the risk of B[a]P in the soil environment.

## Methods

### Chemical and biological materials

The B[a]P standard (chromatographic reagent grade) and SUPELCOSIL LC-PAH column (25 cm long × 4.6 mm i.d., 5 µm particle size) were obtained from Supelco Company, Bellefonte City, PA, USA. Tenax-TA, a porous polymer of 2, 6-diphenyl furan resin of 60–80 mesh, was purchased from Beijing KangLin Science & Technology Co. Ltd., Beijing, China. Red wiggler worms (*Eisenia fetida*) were selected as the test organism and purchased from a Liu’an earthworm farm in Anhui province, China.

### Soil

#### Soil sample preparation

The soil sample was collected from the High-Tech Demonstration Garden (E117°20′38″, N31°92′43″) of Anhui Agricultural University, China. Soil samples were passed through a 2-mm sieve, and then stored in a glass bottle at 4 °C prior to use. Each kilogram of soil contained 0.69 g total N, 11.78 g organic matter, 61.39 mg available N (alkali-hydrolyzable nitrogen), 10.25 mg available P (Olsen-P) and 175.49 mg available K (NH_4_OAc-K). Soil pH was 6.76. The background concentration of B[a]P in soil was 3.32 µg kg^−1^. The actual status was multiple pollutions, and 12 additions can reach pollution equilibrium and reflect the actual situation of multiple soil pollution.

#### Soil incubation experiments

Two kilograms of air-dried soil and all the glassware were sterilized at 100 °C for 2 h, and then cooled to room temperature prior to the experiment. The multiple-time and one-time additions of B[a]P in soil were performed as follows. First, a high concentration (10.0 mg kg^−1^) of B[a]P-spiked soil was prepared. Briefly, 20 g sterile soil were added to a 100-mL glass beaker containing 20 mL solution of dichloromethane and acetone (1:1, v/v) and 200 µg B[a]P; the mixture was then stirred with a glass rod. The organic solvent residue in the soil was volatilized for 24 h in a fume hood. The concentration of B[a]P in the high-concentration B[a]P-spiked soil was 10.0 mg kg^−1^. Then, the additions of B[a]P-spiked soil into uncontaminated soil were carried out using two different approaches (one-time or multiple-time additions). In the multiple-time addition method, 10 g of freshly-prepared high B[a]P-spiked soil (10.0 mg kg^−1^) was thoroughly mixed with 880 g of soil, to produce the working B[a]P concentration of 100 µg kg^−1^ soil. At two-week intervals, a further subsample of 10.0 mg kg^−1^ B[a]P-spiked soil was added to the previously B[a]P-treated soil following the procedure described above. At each addition, the concentration of B[a]P in the soil increased by 100 µg kg^−1^. After 12 additions, the final concentration at week 22 of B[a]P in the multiple-time addition treatment was 1200 µg kg^−1^. In the one-addition treatment, 120 g of soil with 10.0 mg kg^−1^ of B[a]P were added into 880 g of soil at one time, and the final concentration of B[a]P in one-time addition was 1200 µg kg^−1^. The treatments without B[a]P were designated as the controls. Each treatment was carried out three times.

After the addition treatments were completed, the soil samples prepared by one-time or multiple-time addition methods were incubated for 56 d at 25 °C. Soil moisture content was maintained at 75% of the water-holding capacity, using deionized water.

### Extraction of the available fraction of B[a]P in soil

For the extraction of the soil-available fraction of B[a]P, a 2.0-g soil subsample, which was taken from each replicate of the one-time or multiple-time addition treatments at the end of each of the five incubation periods (1, 7, 14, 28 or 56 d), was placed in a 100-mL conical flask with 70 mL 0.01 M CaCl_2_ solution. Tenax-TA beads (0.2 g) were added to the flask, which was continuously shaken end-over-end on a shaker at 100 rpm at room temperature (20 ± 2 °C) for 6 h^35^. Afterwards, the Tenax-TA beads were rinsed with 20 mL deionized water, and then the B[a]P was extracted from the beads with 20 mL (4 × 5 mL) acetone on a tumble shaker at 50 rpm for 5 min. The pooled extracts from the Tenax-TA beads were partitioned with a 15-mL mixture of petroleum ether and dichloromethane (9:1, v/v). The solvent layer was concentrated to 2 mL for further purification. Each soil replicate was extracted by Tenax-TA in triplicate.

### Earthworm incubation

Red wiggler worms (*Eisenia fetida*), which were of similar age, individual weight (0.4–0.5 g, fresh weight), and length (5–7 cm), with a full developed clitellum, were used in this study. After being rinsed with deionized water, they were incubated in a glass dish with clean moist filter paper at 20 ± 2 °C for 24 h in a dark room to void their gut contents.

Each soil treatment was destructively sampled and sterilized^7^ after incubation for 1, 7, 14, 28 or 56 d. Six earthworms were introduced into a 100-mL beaker with 60 g sterilized soil at each of the different periods. The earthworms were incubated in the beakers for 7 days at 20 °C (with the soil being maintained at 50% maximum water-holding capacity using deionized water), and no mortality occurred during the entire incubation period. Earthworm tissue samples were taken immediately at the completion of the 7-d incubation period for extraction of B[a]P.

### Extraction of B[a]P in earthworms

After 7 d incubation in the soil, the earthworms were removed from the flasks. Their guts were evacuated by incubation on clean moist filter paper at 20 ± 2 °C for 48 h^36^. The extraction of B[a]P in earthworms was performed by accelerated solvent extractions (ASE300, Dionex, Sunnyvale, CA, USA). Earthworm tissue (1 g) was frozen in liquid nitrogen, crushed to a fine powder using a pestle and mortar, and completely mixed with 0.5 g silica and 5 g florisil prior to being transferred to an extraction cell. The remaining space of the cell was filled with clean quartz sand. The top and bottom ends of the extraction cell were covered with a piece of cellulose filter paper (19.8 mm diameter). The extraction was performed with dichloromethane/acetone (1:1, v/v) at 100 °C and 10.5 MPa by heating for 5 min, followed by 5 min static extraction. Each extraction cell was rinsed with 60% cell volume of the extraction solvent. Then, the extracts were purged from each extraction cell for 60 s using pressurized nitrogen at 10.5 MPa. For each sample, the static extraction, rinsing and purging steps were performed twice and the extracts from each step were pooled. The pooled extract was concentrated to 2 mL using a rotary evaporator, and then partitioned against 15 mL petroleum ether/dichloromethane (9:1, v/v) solvent, and the solvent layer was collected and concentrated to 2 mL.

### B[a]P purification and HPLC determination

The concentrated Tenax-TA and earthworm extracts were purified in a solid-phase extraction (SPE) column (PAH C18 waters). A SPE column (5 mL) was successively filled with three layers: the bottom layer consisted of anhydrous sodium sulfate (1.0 g), the middle layer Al_2_O_3_ (0.5 g) and the top layer florisil (1.5 g). The column was conditioned with 10.0 mL petroleum ether before sample loading. The concentrated extracts (2 mL) were passed through the column at a flow rate of 1.0 mL min^−1^. Subsequently, the column was eluted with 20 mL (4 × 5 mL) solution of dichloromethane and petroleum ether (1:9, v/v) at 1.0 mL min^−1^. The eluate was collected and concentrated to about 1.0 mL in a rotary evaporator, and then evaporated to 1.0 mL by a gentle N_2_ stream before HPLC analysis^37^.

The amount of B[a]P in the sample was determined by high-performance liquid chromatography (HPLC) (Waters 601) with Waters 2475 multi Fluorescence Detector (Waters, Milford, MA, USA). The separation of B[a]P was performed with a SUPELCOSIL C18 (octadecyl) phase LC-PAH column (250 × 4.6 mm I.D., 5 µm particle size; Supelco), maintained at 30 °C. The mobile phase was composed of two solvents, acetonitrile (A) and water (B). The linear gradients were started from 50% A and 50% B, increased linearly to 70% A and 30% B over 2 min, then to 100% A over 6 min, and finally the column was eluted isocratically for 8 min. The gradients were returned to 50% A and 50% B over 1 min, and kept isocratic for 9 min. The total run time was 26 min, and the next run delay was 12 min. The flow rate was set at 2 mL min^−1^ and the injection volume was 20.0 µL. Detection was carried out with a Waters 2475 multi Fluorescence Detector at an excitation wavelength of 294 nm and an emission wavelength of 430 nm. The B[a]P concentration in each sample was quantified, using an external standard method. Blanks were also run for each series of samples. The limits of detection for B[a]P by HPLC is 1 ng ml^−1^. The mean recovery rates of B[a]P in soil and earthworm ranged from 81% to 119%, and the relative standard deviation ranged from 4.9% to 10.8%.

### Enzyme assays

The earthworm coelomocytes were extracted by a mixed solution (pH 7.3) of 5% ethyl alcohol (v/v), 95% saline (v/v), containing 2.5 mg mL^−1^ Na_2_EDTA, and 10 mg mL^−1^ guaiacol glycerol. The homogenate of earthworm coelomocytes was centrifuged at 3500 rpm at 4 °C for 15 min, and the supernatant was used for further analysis. The activity of SOD was determined according to the method described by Marklund^38^. One unit (U) of SOD activity was defined as the amount of enzyme required to cause 50% inhibition of the nitroblue tetrazolium photoreduction rate at 25 °C. The activity of peroxidase (POD) was determined according to the method developed by Kochba *et al*.^39^. The reaction was measured by recording absorbance at 420 nm once 0.1 mL supernatant was added. Protein content in earthworm coelomocytes homogenate (μg mg^−1^) was determined by the bicinchoninic acid method^40^. The specific activities of SOD and POD enzymes were expressed as U mg^−1^ earthworm protein.

### Statistical analysis

The means and standard deviations (SD) were calculated using Microsoft Office Excel 2007. Statistical software (SPSS v.17.0) was used for analysis of variance (ANOVA).
